# Determination of the Concentration of Ultrafine Aerosol Using an Ionization Sensor

**DOI:** 10.3390/nano11061625

**Published:** 2021-06-21

**Authors:** Szymon Jakubiak, Przemysław Oberbek

**Affiliations:** Department of Chemical, Aerosol and Biological Hazards, Central Institute for Labour Protection—National Research Institute, Czerniakowska 16, 00-701 Warsaw, Poland; ober@ciop.pl

**Keywords:** nanoparticles measurement, ultrafine aerosol, ionization sensor, environmental monitoring, environmental engineering, exposure assessment, occupational safety and health

## Abstract

As public awareness of the threats related to ultrafine aerosols increases, there is a growing need for inexpensive, real-time exposure assessment devices. In this work, the well-established technology used in the smoke detector with a radioactive source was tested in laboratory conditions to check its suitability for determining the number concentration of the ultrafine aerosol. It has been shown that the sensor output changes linearly with the change of diesel soot concentration in the range up to 8.3 × 10^5^ particles cm^−3^. The sensor has also been shown to be able to detect rapid changes in aerosol concentration. Empirical equations describing the influence of air velocity, temperature, relative humidity, and pressure on the sensor output were determined. The collected results confirm that the ionization sensor can be used to assess ultrafine aerosol exposure, although additional engineering work is required to increase the resolution of the output signal measurement and to compensate for the effects of weather conditions. The presented method can be used for concentration monitoring and risk assessment in environmental engineering, materials engineering, cosmetics industry, textiles, sports, chemical, mining, energy, etc.

## 1. Introduction

Engineered nanoobjects, their agglomerates, and aggregates (NOAA) can exhibit properties that are different from their non-nanoscale (bulk) material equivalent [1,2,3,4,5]. The matter at the nanoscale obtains new chemical, biological (including toxicological), mechanical, and physical properties with enhanced performance over their micro counterparts [6]. Nanomaterials have found a broad range of applications in environmental engineering, materials engineering, health, mining, aerospace, sports, and various other industries [5,7].

In the work environment, ultrafine aerosols can be created spontaneously as a byproduct (e.g., due to combustion processes or welding) or due to the emission of nanoparticles from technological processes such as grinding, crushing, polishing, or transporting. Moreover, engineered nanomaterials can be used as substrates or additives in many different technological processes [8].

Inhalation is considered to be one of the main routes of human exposure to nanoobjects [9]. Nanomaterials are a very diverse group of chemicals; it is thus difficult to make general statements about their potentially adverse health effects. Over the years, studies have been conducted on the effects of NOAA impact on human health using the in vitro models [10,11,12,13]. Besides direct exposure, NOAA can also be accumulated in soil and plants, causing a risk not only to people directly involved in their production or processing [14]. Moreover, it needs to be taken into account that a specific nanocompound (in several forms) may exhibit different biological activity depending on the production method and processing [15,16]. In the case of the risk evaluation for workers handling particular nanomaterials (and their different forms), it is easiest to begin with exposure assessment to NOAA in the air [7,17]. Based on this assessment, occupational health and safety specialists can take appropriate steps if necessary. Currently, there is an absence of health-based regulatory occupational exposure limits (OELs) for various NOAA. There are also no specific international regulations, international protocols, or legal definitions for the production, handling, or labeling of NOAA. However, there are general rules and recommendations to protect the workforce proposed by Organization for Economic Cooperation and Development (OECD), World Health Organization (WHO), and ISO [18,19,20,21,22]. The WHO has adopted similar threshold values to nano reference values (NRV) for nanomaterials based on the number concentration and density of NOAA proposed by the Social and Economic Council (SER, Sociaal-Economische Raad), the advisory body to the Dutch government and the parliament on national and international social and economic policy [23,24,25]. The NRVs are limit values whose exceeding should result in the application of appropriate exposure control measures. Generally, the threshold for metallic particles is 20,000 particles cm^−3,^ and for metal oxides, carbon black, fullerenes, dendrimers, polystyrene, etc. is 40,000 particles cm^−3^. These are temporary values and may change as knowledge of the toxicity of nanomaterials progresses.

Documents mentioned above listed several direct-reading instruments for counter methods providing real-time measurements of mass concentration (based on assumed nanomaterial density) and the number concentration of NOAA in the air. There are also gravimetric methods mentioned for post-factum mass concentration evaluation (independent of knowledge on nanomaterial density). Moreover, cascade impactors can be used to sample different aerosol fractions down to ultrafine particles [26]. The disadvantages of the counter methods are their relativity (the effect of which is the discrepancy between the indications of two different instruments) and the lack of appropriate methods of calibration [21,27,28]. The disadvantages of gravimetric methods are their low accuracy (which depends on the sensitivity of the scale), long analysis time (depending on weighing procedures, filter conditioning), and susceptibility to physical stimuli (shock, vibrations, handling by an analyst).

There is, however, a great limitation of the risk assessment connected to NOAA exposure in the work environment, which is the general lack of high-quality exposure data [29]. There is no international standard describing how such measurements should be conducted and what devices should be used [30]. Several measurement systems exist capable of in situ and on-line ultrafine aerosol number concentration determination. The most popular are scanning mobility particle sizers (SMPS), combining differential mobility analyzers (DMA) with condensation particle counters (CPC). Slightly less popular are the systems consisting of DMA’s in combination with Faraday cup electrometers and electrical low-pressure impactors. Moreover, there are portable devices capable of personal NOAA exposure assessment using electrometer measurements of charged particles [31,32,33] or oscillation change of the microelectromechanical resonant cantilever [34,35].

The main disadvantage of these systems is the high cost of purchase and operation. As a result, they are only used for periodic measurements, which may not give full information about the NOAA exposure. Therefore, there is a need for low-cost devices capable of NOAA concentration determination. In 2019, a project focused on developing a workplace monitoring strategy for measuring NOAA with low-cost sensors was initiated by CEN. The main focus is on verifying the suitability of the available low-cost environmental sensors for measuring the concentration of agglomerates and aggregates of nanoobjects. Based on a review of literature sources, no low-cost devices capable of measuring the primary nanoobjects concentration were identified.

**Figure 1:**
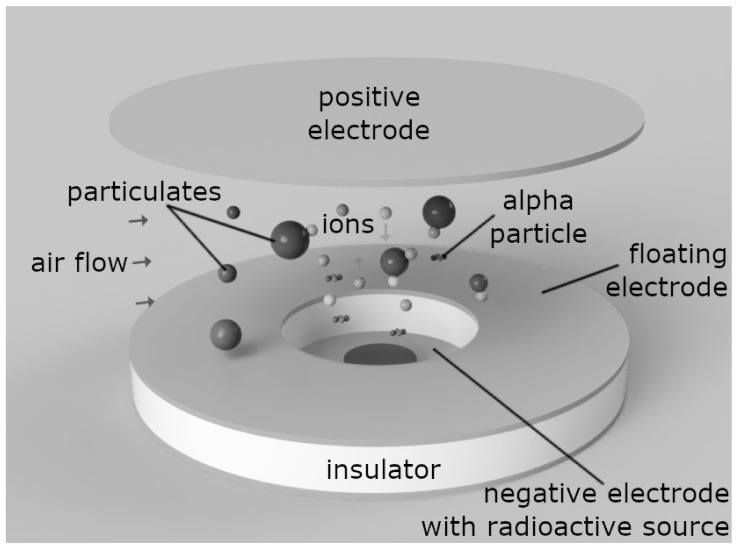
Schematic diagram of the ionization chamber.

Ionization smoke detectors are widely used fire safety devices whose technology dates to the 1950s. Their main advantage over optical sensors is a faster response to flaming fires when the particles created due to combustion are relatively small (in the range from several nanometers to one micrometer). A schematic diagram of the ionization sensor is presented in Figure 1. A small amount of radioactive material (typically americium-241) is placed in the center of a disk, which is the negative electrode. On the top of the bottom disk, there is an insulating material and a floating (or collection) electrode, which is electrically insulated from other components and connected to the electronic circuit capable of electrostatic potential measurement. The volume beneath the floating electrode is referred to as the reference chamber, while the volume above it, bounded from the top by the positive electrode, is referred to as the active chamber. Alpha particles (identical to helium-4 nuclei) emitted from radioactive material ionize the air causing the formation of positive and negative ions. Due to the ions’ transport towards the electrodes with opposite signs, electric currents are generated. If particle-free air is present in the active chamber, the electric currents in both chambers are equal and the electrostatic potential of the floating electrode is in equilibrium. If particulates enter the active chamber, some of the alpha particles and generated ions attach to their surface, causing a change in the electrostatic potential measured on the floating electrode. In the case of the smoke detector, this change is compared with the threshold value and used to trigger the alarm.

The application of modified smoke detectors for particulate matter sensing has been previously proposed by several authors. Litton et al. [36] applied both optical and ionization smoke detectors for concentration measurements of micrometer and submicrometer aerosols. This idea was then developed by Edwards et al. [37]. It was shown that the ionization sensor was approximately five times more sensitive to the presence of fine particles, while the photoelectric sensor was about five times more sensitive to the presence of coarse particles. The lower detection limit for fine particles was estimated to be at the level of 17 µg m^−3^. Moreover, it was stated that besides particle concentration, environmental conditions such as temperature, humidity, and pressure will also influence the measured signal value. Dahl et al. [38] proposed an application of the modified ionization smoke detector as a low-cost nanoparticle monitor. To overcome the influence of environmental conditions on the output signal, measurements were conducted for filtered and untreated air. The lower detection limit for 100 nm particles was estimated at the level of 15,000 particles cm^−3^. The tested sensor showed a linear response for concentration change of KCl calibration aerosol, candle smoke, and welding fumes.

In the present paper, the possibility of measuring the concentration of primary nanoobjects and their agglomerates and aggregates using an ionization sensor has been verified. Response of the ionization sensor to NOAA concentration change was investigated in laboratory conditions. Artificial diesel soot aerosol was chosen as a test medium. Diesel soot is a common pollutant that can be found in outdoor air and at workplaces, which is known to be a carrier for toxic and carcinogenic substances [39]. Moreover, the influence of the air temperature, relative humidity, pressure, and velocity on the value of the output signal was determined.

## 2. Materials and Methods

**Figure 2:**
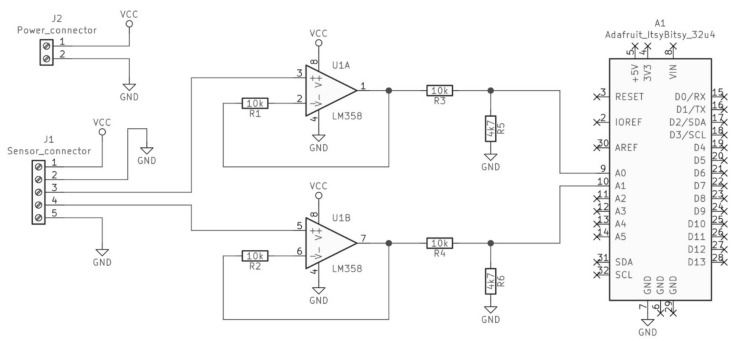
Diagram of ionization sensor signal processing circuit.

The ionization sensor from a smoke detector DIO-40 (POLON-ALFA, Bydgoszcz, Poland was modified by exposing two signals: the supply voltage (*V_sup_*) and the voltage proportional to the electrostatic potential measured on the floating electrode (*V_mea_*). Both signals were processed by the operational amplifiers LM358 (gain equal 1) and measured using a microcontroller ItsyBitsy 32u4 (Adafruit, New York City, NY, USA) with 10-bit analog-digital converter (ADC) through voltage dividers (10 kΩ/4.7 kΩ). As the ionization sensor was powered by 12 V DC, voltage dividers were necessary to shift signals to the level safe for the ADC (0–5 V). The voltage resolution of the ADC was approximately 0.005 V. The electric diagram of the signal processing circuit is presented in Figure 2.

To investigate the output signal stability, the sensor was mounted in a laminar flow cabinet. Raw sensor output values were read by the microcontroller with the frequency of 50 Hz and sent to the PC. Measurements were conducted without airflow and with an airflow of 0.5 m s^−1^.

**Figure 3:**
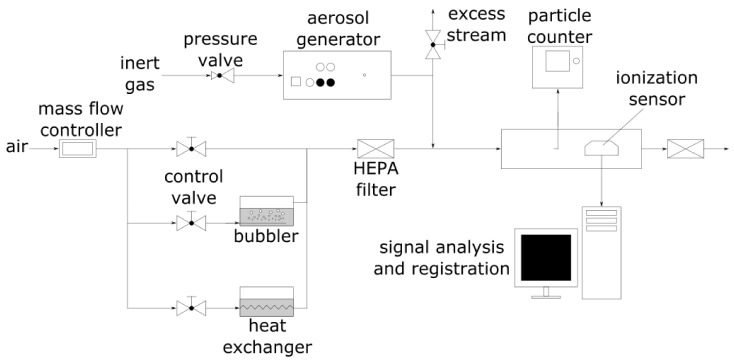
Schematic of the test stand used to determine the influence of the environmental conditions on the ionization sensor output signal value.

The schematic of the test setup is presented in Figure 3. To examine the ionization sensor output in different atmospheric conditions, it was mounted in a pipe with a cross-section of 100 cm^2^. Airflow was generated using HEPA filtered air from the central compressed air system. The air velocity was measured by an IRIS 100 damper connected to a P26 (Halstrup Walcher, Kirchzarten, Germany) differential pressure transmitter sending the measured values to the PC. The air velocity was kept constant at 0.15 m s^−1^ during all tests except when its influence on the sensor output was examined. Value of the output signal *V_out_* was calculated every second directly on the microcontroller according to Equation (1):

(1) $$V_{out}={\bar{V}}_{sup}-{\bar{\;V}}_{mea}$$

where ${\bar{V}}_{sup}$ and ${\bar{V}}_{mea}$ are the average values of the supply voltage and voltage proportional to the electrostatic potential measured on the floating electrode, respectively, calculated from 50 consecutive measurements.

The test aerosol consisted of ultrafine particles (carbon soot) generated using PALAS GFG 1000 spark discharge generator in an argon atmosphere (4 LPM, 2 sparks s^−1^) diluted with air filtered and dehumidified using 3074B (TSI, Shoreview, MN, USA) air supply unit. Aerosol concentration was controlled by a throttle valve placed on the excess stream from the aerosol generator. Particle size distribution was determined using NanoScan SMPS Model 3910 (TSI, Shoreview, MN, USA) condensation particle counter with 13 channels in a size range from 10 to 420 nm. According to the user manual, the device is capable of measuring the aerosol particle concentrations in the range of 10^2^–10^6^ particles cm^−3^. A schematic diagram of the test setup is presented in Figure 3.

To determine the influence of air temperature on the sensor output, a coil pipe immersed in a heating/cooling medium was used. To elevate humidity, part of the air stream was passed through a container with heated distilled water. The influence of air pressure on the sensor output was examined in a sealed container filled with air filtered and dehumidified using TSI 3074B air supply unit. Air temperature, relative humidity, and pressure were measured using BME280 (Bosch Sensortec, Reutlingen, Germany) sensor connected to a microcontroller Uno (Arduino, Turin, Italy) and registered on the PC.

To assess the possibility of detecting rapid concentration changes, a comparison test with the AeroTrak 9000 (TSI, Shoreview, MN, USA) was conducted. The AeroTrak 9000 is a nanoparticle aerosol monitor that measures the surface area concentration of particles by sensing the charge of particles using an electrometer. In contrast to the TSI NanoScan SMPS, data are acquired with a resolution of 1 s allowing the detection of rapid concentration changes. Both the ionization sensor and AeroTrak were installed under the fume hood (the ventilator was turned off during the measurements). The test aerosol was generated using the GFG 1000 (Palas, Karlsruhe, Germany) generator with graphite electrodes. The spark frequency setting was used to change the aerosol concentration (1 Hz, 5 Hz, 15 Hz, 30 Hz, 60 Hz, 120 Hz), while the inert gas (Ar) flow was kept at the constant level of 4 LPM. The AeroTrak monitor was set to measure the surface area concentration of particles that deposited in the alveolar region of the respiratory tract. In this experiment, there was no forced airflow through the ionization sensor chamber, therefore, its operation was similar to a typical smoke detector application.

## 3. Results

### 3.1. Output Signal Stability

Results from the output stability test are presented in Table 1. Without any airflow present, the measured voltage values were equal to 3.400 and 2.248 in the case of the supply line and output from the floating electrode, accordingly. Although a slight decrease in both signals was observed under laminar flow conditions, its value, as well as the values of the standard deviations, were comparable with the voltage resolution of the applied ADC.

**Figure 4:**
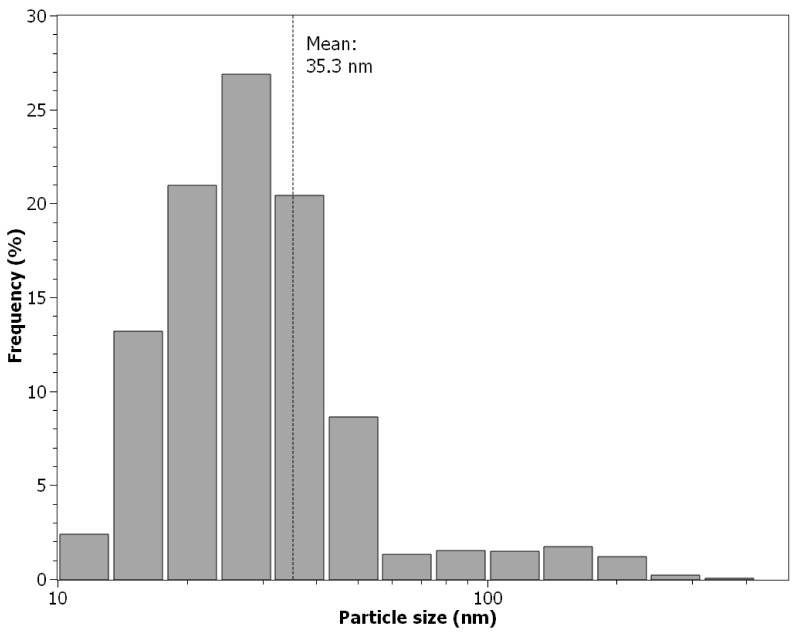
Size distribution of the test aerosol.

Aerosol particle size distribution is presented in Figure 4. It has a lognormal distribution with a maximum frequency peak of 26.9% for particles with a size of 27.4 nm and a mean size of 35.3 nm.

### 3.2. Response to the Aerosol Concentration Change

**Figure 5:**
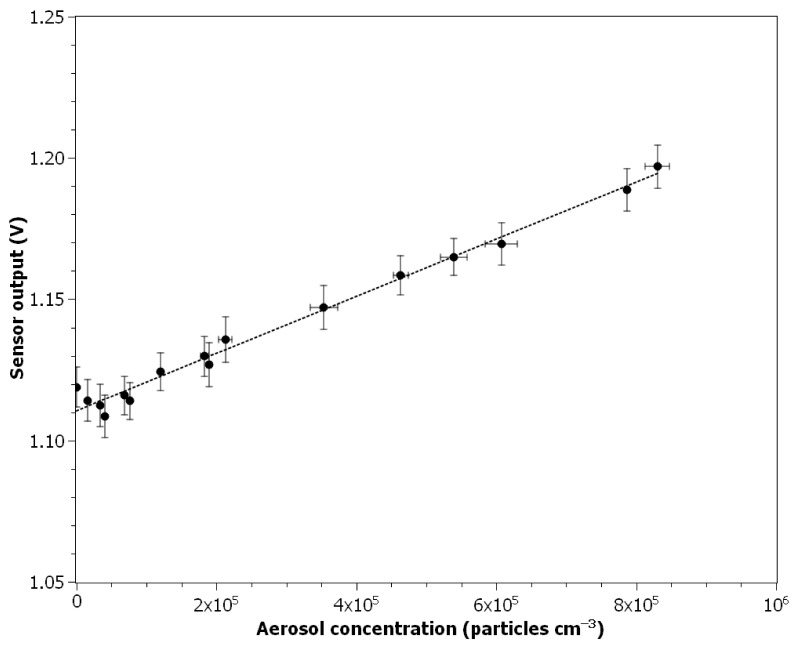
Dependence between ionization sensor output and ultrafine aerosol particles concentration.

Figure 5 demonstrates the ionization sensor output in response to ultrafine aerosol concentration changes. Each data point is calculated as an average from 3 min of measurements (3 measurements performed by NanoScan SMPS and 180 measurements performed by the ionization sensor). Sensor output increases linearly with the aerosol concentration increase, which can be explained by the decrease of *V_mea_* due to the bonding of ions generated in the chamber with the surface of aerosol particles. The relationship can be approximated for the measured aerosol concentration up to 8.3 × 10^5^ particles cm^−3^ by the following equation (coefficient of determination *R*^2^ = 0.986):

(2) $$V_{out}=1.010\times 10^{-7}\times c_{n}+1.111$$

where *c_n_* is the number concentration of aerosol particles in particles cm^−3^. The value of the Pearson correlation coefficient calculated for 48 data points (1-min averages) was equal to 0.990. Air parameters during measurements: temperature: 25.5 ± 0.1 °C, relative air humidity: 0%, pressure: 100.70 kPa, velocity: 0.15 ± 0.02 m s^−1^.

### 3.3. Response to the Air Velocity Change

**Figure 6:**
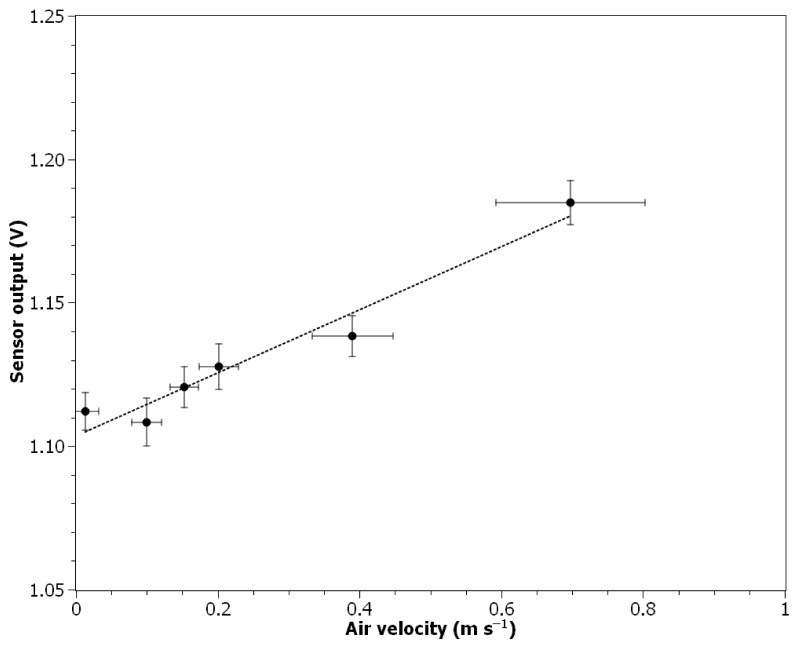
Ionization sensor output signal change in relation to air velocity.

The ionization sensor used in a smoke detector is designed to work in the absence of any viscous drag forces. The influence of air velocity on the sensor output is presented in Figure 6. It can be seen that the sensor output increases with increasing air velocity (leaching of the ions from the measurement chamber causes the decrease of *V_mea_*) and the relationship can be approximated in a measured air velocity range by linear regression (*R*^2^ = 0.955):

(3) $$V_{out}=0.110\times U+1.104$$

where *U* is the air velocity in m s^−1^. The value of the Pearson correlation coefficient calculated for 18 data points (1-min averages) was equal to 0.973. The average air parameters during experiment: temperature 26.2 ± 0.1 °C, relative humidity: 3.7 ± 0.1%, pressure: 100.77 kPa. The average concentration of particles was below 10^2^ particles cm^−3^ specified as the lower detection limit for the counter used.

### 3.4. Response to the Relative Air Humidity Change

**Figure 7:**
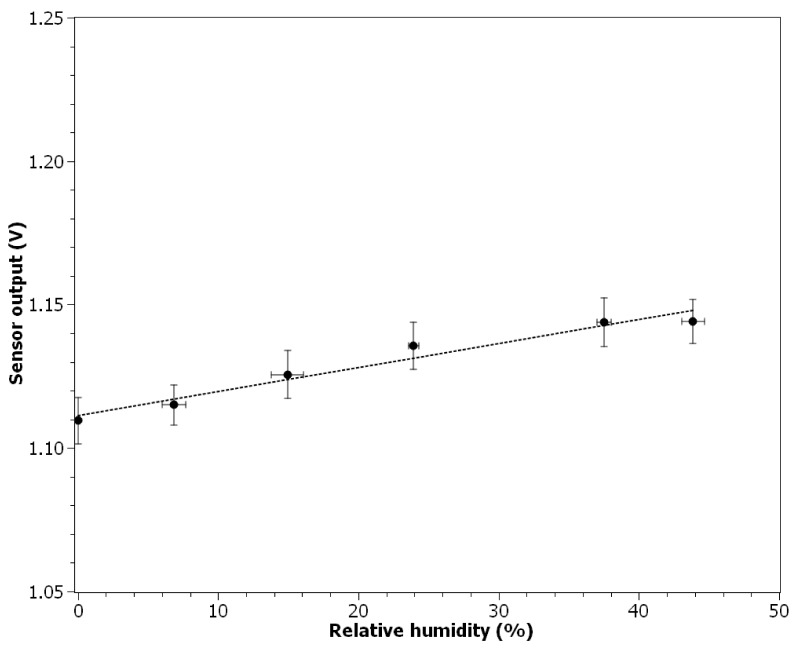
Relation between ionization sensor output and relative air humidity.

Figure 7 presents the sensor output response in relation to the relative air humidity change. In the humidity range from 0 to 43.9% the relation was linear (*R*^2^ = 0.958):

(4) $$V_{out}=0.083\times 10^{-2}\times \;H\;+1.111$$

where H is the relative air humidity in %. Higher values of the humidity could not be tested due to the limitation of the particle counter used. The value of the Pearson correlation coefficient calculated for 21 data points (1-min averages) was equal to 0.960. The average air temperature during the experiment was equal to 27.2 ± 0.1 °C, and the average concentration of particles was below 10^2^ particles cm^−3^. The average air velocity was 0.15 ± 0.02 m s^−1^.

### 3.5. Response to the Air Temperature Change

**Figure 8:**
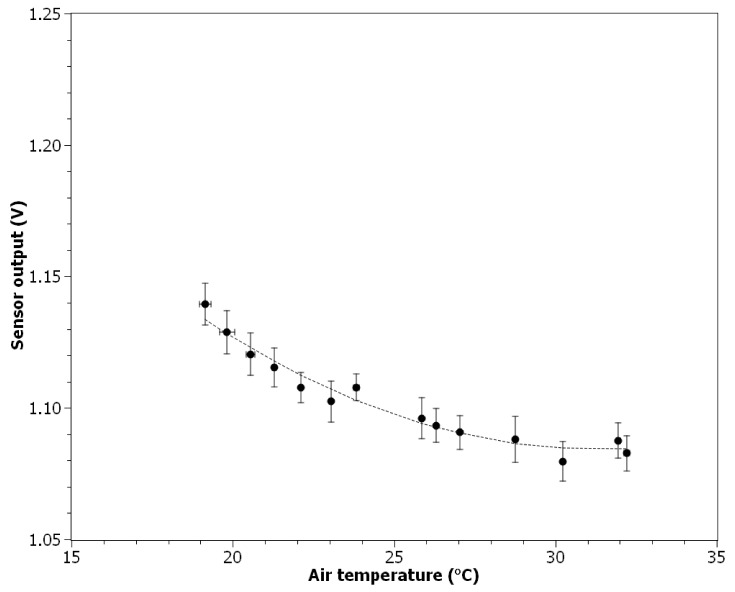
Ionization sensor output signal change in relation to air temperature.

The influence of the air temperature change on the ionization sensor output signal is presented in Figure 8. In the measured range from 19.2 to 32.2 °C the sensor output decreases with air temperature increase. The relationship can be approximated by a polynomial equation (*R*^2^ = 0.951):

(5) $$V_{out}=0.334\times 10^{-3}\times T^{2}-2.091\times 10^{-2}\times T+1.412$$

where *T* the is the air temperature in °C. The value of the Pearson correlation coefficient calculated for 14 data points (15-s averages) was equal to −0.928. Relative air humidity was equal to 0% during this experiment. The average concentration of particles was below 10^2^ particles cm^−3^. The average air velocity was 0.15 ± 0.02 m s^−1^.

### 3.6. Response to the Air Pressure Change

**Figure 9:**
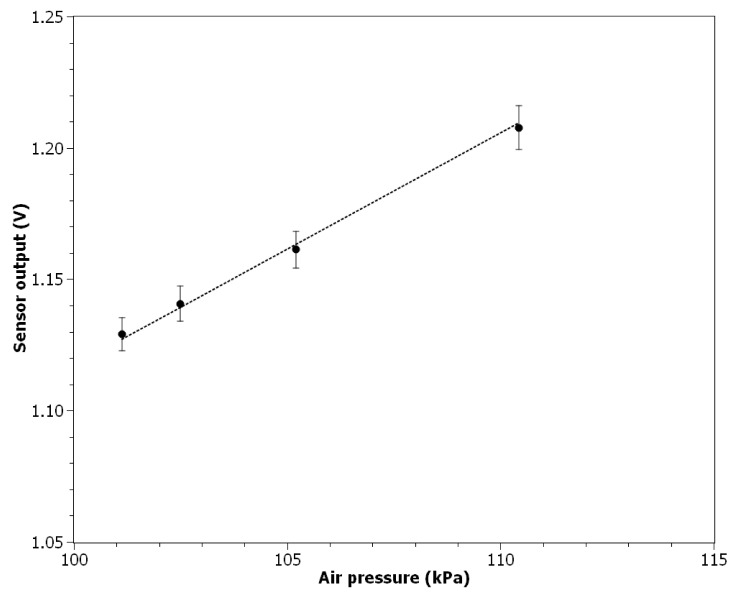
Sensor output signal in response to air pressure change.

The air pressure influence on the sensor output is presented in Figure 9. The measured voltage at the sensor output increases linearly with increasing air pressure. The relation can be approximated by a linear regression function (*R*^2^ = 0.999):

(6) $$V_{out}=8.857\times 10^{-3}\times P+0.231$$

where *P* is the air pressure in kPa. The value of the Pearson correlation coefficient calculated for 7 data points (1-min averages) was equal to 0.999. The average air parameters during the experiment: temperature 22.7 ± 0.1 °C, relative humidity: 3.6 ± 0.2%. The average concentration of particles was below 1 × 10^2^ particles cm^−3^. Measurements were conducted without airflow.

### 3.7. Ability to Detect Rapid Aerosol Concentration Changes

**Figure 10:**
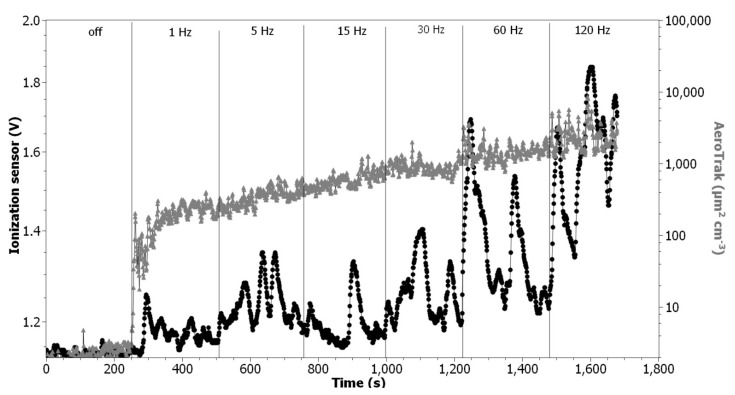
Comparison of ionization sensor and TSI AeroTrak 9000 responses to diesel soot concentration changes.

In Figure 10, the ionization sensor output is compared with the results of the surface area concentration measurements conducted using TSI AeroTrak 9000. Peaks in the output values can be observed for both sensors, although they were broader and slightly shifted to the right in the case of the ionization sensor due to the lack of forced airflow through the measurement chamber. Moreover, good agreement occurred in the presence of the peaks in the whole aerosol concentration range. The Pearson correlation coefficient calculated for 1679 data points was 0.77, which means that there is a strong correlation between the output values from both sensors. It can also be seen that the amplitude of signal peaks increases with the increasing sparking frequency setting of the GFG 1000 generator.

The average air parameters during experiment: temperature 25.2 ± 0.3 °C, relative humidity: 49.3 ± 0.8%, pressure: 100.73 ± 0.02 kPa.

## 4. Discussion

The calculated value of the output signal noise level of 0.010 V agrees with the value provided by Litton et al. [36]. According to Equation (2), this converts into a difference of ±1 × 10^5^ particles cm^−3^ in aerosol concentration measurements. Such a noise level seems too high for the planned application, although as it can be seen in Figure 5, by averaging the measurement results in 3-min time frames, it was possible to achieve a strong linear correlation with the reference device. To decrease the noise level several hardware modifications could be made. The microcontroller used in this experiment has a 10-bit precision ADC. This means that the voltage values from 0 V to 5 V are read as digital values from 0 to 1023, accordingly. Therefore, a change of the measured voltage by 0.010 V results in a change of digital value by 2. By incorporating 12-bit precision ADC, where 5 V is read as a value of 4095, the same voltage change value will result in a change of digital value by 8.

During the stability test, a slight difference was observed in the mean voltage values measured in the reference chamber without the airflow and under the laminar flow conditions, although the minimum and maximum measured values were the same (Table 1). In the conducted experiments, a USB power line from the PC was used as a 5 V reference value for ADC. Although since the output signal from the ionization sensor was calculated as a difference between the two measured voltages, this should have no impact on the measurement accuracy, application of a high precision external reference voltage source could improve the output signal stability.

Presented experimental results showed that the output signal from the modified ionization sensor used in the smoke detector could be applied to ultrafine aerosol particle number concentration determination. As it can be seen in Figure 5, the mean values of ionization sensor output signal measurements are well aligned in a linear relationship with the aerosol number concentration determined using the NanoScan particle counter, which is in good agreement with the results presented by other authors [36,38]. It can be concluded that the measuring range of the nanoobject sensor using the ionization detector will be similar to that of other devices based on the electrometric principle of measuring the concentration of nanoaerosols. The Grimm MiniWRAS particle counter has a measuring range of 3000 to 500,000 particles cm^−3^. The Testo DiSCmini portable counter has a measuring range of 1000 to 1,000,000 particles cm^−3^, as does the NanoScan counter used as a reference device. It can also be noted that the calculated standard deviations of the sensor output signal mean values are similar in the whole measured particle concentration range, while in the case of the reference device (TSI NanoScan SMPS), the standard deviation increases with increasing concentration. This is probably due to the coincidence error, which is a typical problem in the case of optical particle counters.

From a comparison test with the TSI AeroTrak 9000, it can be seen that the ionization sensor can respond quickly and with high sensitivity to the rapid changes in aerosol concentration (Figure 10). The amplitude of the ionization sensor output signal peaks is well aligned with the amplitude of the peaks registered using AeroTrak monitor, although they differ in width due to the previously mentioned reason. It should be noted that there is a difference in the way the output signal is generated for these devices. As already mentioned, in the case of the ionization sensor, the change in the electrostatic potential between the electrodes is recorded as a result of a decrease in the number of charge carriers. In the case of the AeroTrack sensor, the particles are charged with hydrated protons generated by the corona discharge. The output signal is then generated by passing the positively charged particles through an electrometer.

It needs to be noted that the presented results of comparison tests with commercial nanoparticle sensing devices are valid only for the specific test aerosol used. Other particulates may differ in surface parameters such as charge value and distribution, specific area, or affinity to water, which may affect how they will interfere with ions in the active chamber. This entails the need for calibration for a specific type of particulate, optimally carried out in the place where the sensor will be used. Alternatively, a diffusion charger could be applied similarly, as it was done, for example, in the TSI AeroTrak, although this would greatly increase the device complexity.

To build a measuring device using the tested ionization sensor, some additional engineering work needs to be done. To ensure that only ultrafine particles are being measured, an impactor with the appropriate cutoff point needs to be designed and installed on the inlet [40]. One of the inputs for the impactor design is the air velocity, which determines the value of the inertial force acting on particles. Besides the previously mentioned issue with the leaching of ions from the measurement chamber, it needs to be noted that precise control of airflow is important in all types of particle counters—both the number of particles and volume of the sampled aerosol are needed to determine the concentration. Therefore, it is important to implement a closed-loop control system allowing for maintaining the air velocity at a constant level. Although, as shown in Figure 6, the standard deviation of air velocity measurements conducted with the IRIS 100 damper increases proportionally to its value, it does not seem to affect the stability of the ionization sensor output. However, it can be expected that the formation of vortexes at higher air velocities may affect the sensor performance. It seems that the value of 0.1–0.2 m s^−1^ should be a good compromise between the response time of the sensor and its stability.

Perhaps the biggest challenge in using an ionization sensor to determine nanoparticles concentration will be to account for the influence of air parameters. This problem also occurs with the current state-of-the-art devices, e.g., those using electrometers. In theory, the available low-cost temperature, relative humidity, and pressure sensors (which may have rice grain size) could be used to account for changes in these parameters with respect to the calibration conditions (Equations (4)–(6)). While this may be possible for pressure and temperature (not trivial due to the polynomial relationship), it is questionable in the case of relative humidity. Moisture present in the air will influence both the electrical parameters of the air and the surface charge of the particulates, which may influence how ions in the active chamber interfere with them. To overcome this issue without an excessive increase of technical complexity (thus device cost), a resistive heater made of conductive PTC rubber may be applied to avoid operation under moisture condensation conditions. Moreover, it needs to be noted that obtained polynomial relationship between the air temperature change and the ionization sensor output is in contrast with the results presented by [37], where a linear equation was proposed to fit the results obtained using the theoretical model.

Alternatively, particle concentration measurements can be performed by comparing the outputs from the ionization sensor measured for the sampled aerosol and HEPA filtered air. In this case, additional tests should be carried out to confirm that the relationship between the output signal value and the aerosol concentration presented in Figure 5 is similar regardless of the air parameters.

## 5. Conclusions

Based on the literature review and experimental results, it was demonstrated that the ionization sensor from a commonly used smoke detector could be used to roughly determine the concentration of the ultrafine aerosol. Due to the widespread adaptation, such sensors are easy to source and low-cost. This makes it possible to build a low-cost device for continuous monitoring of the concentration of ultrafine particles. Currently available devices capable of performing such measurements, due to the high purchase and maintenance costs, are used only for periodic measurements. Continuous air quality monitoring is important for processes where nanoparticles are used as substrates or additives, but also for processes where ultrafine particles may be released as a result of an accident or failure. This will allow to improve work safety and reduce costs related to accidents and other unforeseen events by enabling quick detection.

## Figures and Tables

**Table 1:** Stability of the raw sensor output signals during 20 s of measurements with a frequency of 50 Hz.

Conditions	Supply Voltage (V)				Floating Electrode (V)			
	Avg.	St. Dev.	Min.	Max.	Avg.	St. Dev.	Min.	Max.
without air flow	3.400	0.007	3.382	3.407	2.248	0.010	2.224	2.268
laminar flow 0.5 m s^−1^	3.397	0.008	3.382	3.407	2.243	0.006	2.219	2.258

## Data Availability

Data available on request from the corresponding author.
